# Occurrence and Characteristics of *Staphylococcus aureus* in a Hungarian Dairy Farm during a Control Program

**DOI:** 10.3390/pathogens10020104

**Published:** 2021-01-21

**Authors:** Flóra M. Petróczki, Ákos Pásztor, Kata D. Szűcs, Károly Pál, Gábor Kardos, Ervin Albert, Brigitta Horváth, Erika Ungvári, Béla Béri, Ferenc Peles

**Affiliations:** 1 Faculty of Agricultural and Food Sciences and Environmental Management, Institute of Food Science, University of Debrecen, 4032 Debrecen, Hungary; pal.karoly@agr.unideb.hu (K.P.); pelesf@agr.unideb.hu (F.P.); 2 Faculty of Agricultural and Food Sciences and Environmental Management, Doctoral School of Animal Science, University of Debrecen, 4032 Debrecen, Hungary; horvath.brigitta920108@gmail.com; 3 WESSLING Hungary Ltd., 1045 Budapest, Hungary; pasztor.akos@wessling.hu; 4 Pázmány Péter Catholic University, 1088 Budapest, Hungary; szucs.katad@gmail.com; 5 Faculty of Medicine, Department of Medical Microbiology, University of Debrecen, 4032 Debrecen, Hungary; kg@med.unideb.hu; 6 Department of Pathology, University of Veterinary Medicine, H-2225 Üllő, Hungary; albert.ervin@univet.hu; 7 Department of Animal Science, Faculty of Agricultural and Food Sciences and Environmental Management, University of Debrecen, 4032 Debrecen, Hungary; beri@agr.unideb.hu; 8 Department of Bacteriology, Mycology and Parasitology, National Public Health Center, 1097 Budapest, Hungary; ungvari.erika@nnk.gov.hu

**Keywords:** antibiotic resistance, bulk milk, enterotoxin, microbiology, *spa* typing, *Staphylococcus aureus*

## Abstract

In this research, our aim was to assess the occurrence of *Staphylococcus aureus* in a Hungarian large-scale dairy farm during the *S. aureus* control program conducted in the course of our studies. Furthermore, the phenotypic and genotypic properties of the isolates (type of haemolysis, antibiotic susceptibility, staphylococcal enterotoxin (SE) gene carrying ability and *spa* type) were determined. *S. aureus* was detected in all bulk tank milk samples collected during this study. Two different *spa* types were identified among the 17 strains isolated in the farm. A total of 14 of the 17 studied strains (82%) showed β-haemolysis on blood agar, 2/17 strains (12%) expressed double zone and 1/17 strains (6%) showed weak β-haemolysis. All strains were susceptible to most antibiotics tested (cefoxitin, chloramphenicol, clindamycin, erythromycin, gentamicin, tetracycline and trimethoprim/sulphamethoxazole), but all strains were resistant to penicillin G. A total of 11 of the 17 strains (65%) were found to harbour *seg*, *sei*, *selm*, *seln*, *selo* genes; 4/17 strains (24%) harboured *sei*, *selm*, *seln*, *selo* genes and 2/17 strains (11%) harboured *sei* gene. Since the new SEs/SEls can also cause foodborne outbreaks potentially and all strains were found to be resistant to penicillin G, it is essential to decrease and keep the prevalence of *S. aureus* low in the dairy farm and the implementation of the *S. aureus* control program is also highly justified. The results showed that the *S. aureus* count decreased by the end of our studies, so the control program was proved to be effective.

## 1. Introduction

Currently, 53 validly published species and 24 subspecies can be distinguished within the genus *Staphylococcus* [1]. Amongst them, *Staphylococcus aureus* is one of the most common pathogens associated with contamination of raw milk and dairy products [2]. *Staphylococcus* spp. are spherical, non-spore-forming Gram-positive bacteria that are facultative anaerobes [3]. Contamination of raw milk with *S. aureus* can occur in the dairy farm, for example, from the skin, mucous membranes of dairy animals, milking equipment, milkers’ hands, or the milking parlour environment [4].

*S. aureus* strains that produce staphylococcal enterotoxins (SEs) can cause food poisoning in human [5]. The number of SEs seems to be growing year by year as more and more types are discovered. Only 14 different SEs were identified in 2003 [6]; according to Benkerroum [7], currently, more than 23 SEs and *Staphylococcus* enterotoxin-like proteins (SEl) can be distinguished. The current nomenclature of SEs uses the name “SE” followed by alphabetic characters in the order of discovery of enterotoxins. The first five SEs (SEA-SEE) are called classical enterotoxins. SEls show significant structural, biological and functional similarities to classical enterotoxins, with the difference that they either do not have the emetic activity characteristic of SEs or further testing is required to determine this [7,8]. SEs and SEls are globular, unique polypeptides with molecular weights ranging from 22,614 to 28,565 Da [3,9]. Genes encoding SEs are carried by mobile genetic elements such as phages, plasmids, and pathogenicity islands. *Staphylococcus* enterotoxin gene clusters (egc) are pathogenicity island-derived structures that also carry SE genes [10]. These toxins are heat resistant and also resistant to some enzymes [6,11,12,13].

Food poisoning is most commonly caused by the classical enterotoxins SEA and SEB, and among the new SEs/SEls by SEH [14,15]. Umeda et al. [16] suggest that new types of SE/SEls may be potential causes of foodborne outbreaks. An analysis of the causes of an outbreak in 2016 revealed, that *S. aureus* isolates that carried the *seg*, *sei*, *sem*, *sen*, *seo*, and *selu* genes without carrying the classical *se* genes may have caused food poisoning. It is, therefore, worth examining not only the genes of classical SEs but also the genes of the new types of SE/SEls. In 2018, a total of 114 outbreaks caused by SEs were reported in the European Union, of which 37 were confirmed outbreaks. None of the total 1124 human cases resulted in death [17].

The *spa* typing is a single locus DNA-sequencing method of the repeat region of the gene (*spa*), encoding protein A, in Staphylococci. This test method is reliable and accurate [18]. A grouping algorithm which is “based upon repeat patterns” (BURP) is recently described and is used for evaluating “the ability of *spa* typing to determine the clonal relatedness of a natural population of *S. aureus* strains” [19].

The α-, β-, γ-, and δ-haemolysins produced by *Staphylococcus* spp. are cytotoxic proteins that damage cell membranes [20] and have the effect of dissolving red blood cells of animal origin. α-haemolysin dissolves red blood cells in sheep and rabbit, and can damage tissue, and the β-haemolysin dissolves red blood cells in cattle and sheep [21].

Antibiotics are used to kill pathogenic microorganisms in the human body. It is important to ensure that the infected organism receives the appropriate amount of the antibiotic in an appropriate duration to prevent the development of resistance [22]. Foods (e.g., milk) that contain antibiotic-resistant microorganisms can be ideal tools for introducing antibiotic-resistant strains into the body [23]. Antibiotic resistance in microorganisms is an important health problem worldwide [24,25]. *S. aureus* is able to adapt to a wide range of environmental conditions and is able to rapidly become resistant to antibiotics [26], for example to β-lactam antibiotics [27].

In this study, our aims were to assess the occurrence of *S. aureus* in a Hungarian large-scale dairy farm and to monitor the phenotypic and genotypic properties of the bacterium (type of haemolysis, antibiotic susceptibility, enterotoxin gene carrying ability and *spa* type) during the *S. aureus* control program conducted in the course of our studies. In terms of the effective control against it, not only the determination of the quantitative data, but also the determination of the characteristics of the bacterium is of great importance.

## 2. Materials and Methods

### 2.1. Place and Date of Samplings

One dairy farm was involved in this study, which was located in the eastern part of Hungary. In the farm, Holstein Friesian cows were milked in milking parlour, and pre- and post-milking disinfection were also used. Total mixed ration (TMR) feeding was applied in the farm, and deep litter was used as keeping method. *S. aureus* control program was applied in the farm, during which, bulk milk samples were collected for our studies between February 2019 and December 2019. In the control program, the stables and the milking parlour were cleaned and disinfected more frequently, the infected cows were isolated and milked separately and their milk was destroyed.

For the microbiological examinations, a total of 35 bulk milk samples were collected in sterile plastic sample tubes from the same milk storage tank containing the milk of all the cows milked in the farm. There were a total of six sampling occasions and 42 to 84 days elapsed between sampling occasions. After sampling, the samples were delivered for analysis to the Microbiological Laboratory of the Institute of Food Science at the University of Debrecen.

### 2.2. Enumeration and Isolation of Staphylococcus aureus

The raw milk sample preparations and the microbiological examinations were performed as described earlier [28]. The bulk tank milk samples, the initial suspension and the tenfold serial dilutions were prepared in accordance with the MSZ EN ISO 6887-1:2017 [29] standard.

The amount of *S. aureus* was determined according to the MSZ EN ISO 6888-1:2008 [30] standard. Baird-Parker agar (BPA) (Biolab Ltd., Budapest, Hungary) was used for the tests, which were supplemented with egg yolk tellurit emulsion (LAB-KA Ltd., Karcag, Hungary). *S. aureus* was differentiated from other *Staphylococcus* spp. with latex agglutination test kit (Prolex^™^ Staph Xtra Latex Kit, Biolab Ltd., Budapest, Hungary). 

Genetic level identification was also performed for each strain; the prevalence of species-specific thermonuclease gene (*nuc*) was tested by PCR method as described below. The primers (Table 1) used for the tests have been previously verified to be specific for *S. aureus nuc* gene [31].

### 2.3. Identification of Staphylococcus aureus with MALDI-TOF MS

As a further confirmation, the strains collected from the samples were identified with MALDI-TOF MS in the microbiological laboratory of the Wessling Hungary Kft in Budapest, Hungary. The *S. aureus* isolates were grown on Columbia Blood agar (Biolab Ltd., Budapest, Hungary) for the tests. A single colony was chosen from the agar and was applied directly to the target plate. One µl of formic acid (70%) was added dropwise to the samples on the target plate and was allowed to dry. Finally, 1 µL of α-HCCA (10 mg/mL) matrix solution was added dropwise to the samples. For the identification of isolates MALDI BioTyper 3.1. Bruker software was used. In the case of the *S. aureus* strain SA57A, SA57B and SA57C the indentification with the MALD-TOF MS was performed at the Department of Bacteriology, Mycology and Parasitology of the National Public Health Center.

### 2.4. Spa Typing

The *spa* typing tests were performed at the Department of Bacteriology, Mycology and Parasitology of the National Public Health Center. The method used by the laboratory is based on the 1.1 version of the description (DNA Sequencing of the spa Gene) of the European Network of Laboratories for Sequence Based Typing of Microbial Pathogens (SeqNet) [32]. BURP clustering for the *spa* types were performed with default parameters with Ridom Staphtype software (Ridom GmbH, Münster, Germany) as described by Mellmann et al. [19].

### 2.5. Haemolysis Testing

Haemolysis tests of collected *S. aureus* strains were performed according to Pereira et al. [33] on Columbia Blood agar.

### 2.6. Antibiotic Susceptibility Testing

The antibiotic susceptibility test of isolated *S. aureus* strains was performed in accordance with the Clinical and Laboratory Standards Institute guidelines [34]. The antibiotic disks applied for the tests were the following: cefoxitin (30 μg/disk), chloramphenicol (30 μg/disk), clindamycin (2 μg/disk), erythromycin (15 μg/disk), gentamicin (10 μg/disk), penicillin G (10U), tetracycline (30 μg/disk) and trimethoprim/sulphamethoxazole (1.25 + 23.75 μg/disk) (Biolab Ltd., Budapest, Hungary). Reference strain ATCC 25923 was used as control.

### 2.7. PCR Amplification of Staphylococcal Enterotoxin-Encoding Genes and Thermonuclease Gene

The PCR tests were performed according to Bianchi et al. [35], with some modifications in the protocol.

Reference strains ATCC 29213 (*sea*; *seg*; *sei*), ATCC 14458 (*seb*), ATCC 19095 (*sec*; *seg*; *seh*; *sei*), ATCC 23235 (*sed*; *seg*; *sei*; *sej*), ATCC 27664 (*see*) were used as positive control. A strain harbouring *selm*, *seln*, *selo* genes isolated from bulk milk was used as reference strain for the test of these genes.

Extraction of genomic DNA from *S. aureus* strains was performed with PrepMan™ Ultra Sample Preparation Reagent (Biocenter Ltd., Szeged, Hungary), according to the instructions of the producer.

For PCR tests, seven primer sets were prepared: Set 1. was designed to amplify *sea*, *seb*, *sec*, *sed*, *see*; Set 2. was designed to amplify *seg*, *sei*; Set 4. was designed to amplify *selm, selo*; Set 6. was designed to amplify *seh* and *ser*. Simplex PCR was used to detect *sej* (Set 3.) and *seln* (Set 5.) enterotoxins and the *nuc* gene (Set 7.). The primer sequences and amplicon sizes are shown in Table 1.

DNA amplification was carried out in a T100™ Thermal Cycler (Bio-Rad Hungary Ltd., Budapest, Hungary). The following amplification cycles were used: the initial denaturation was at 95 °C for five min, followed by 35 amplification cycles (denaturation at 95 °C for 30 s, annealing at 52 °C (56 °C for *nuc*) for 30 sec, extension at 72 °C for one min). The final extension was at 72 °C for 10 min. The amplified samples were analysed by electrophoresis at 120 V for 40 min with PowerPac Basic power supply (Bio-Rad Hungary Ltd., Hungary). One percent agarose gel (Lab Mark Ltd., Praha, Czech Republic) was used, and 5 µL/1000 mL Midori Green Advance dye (Nucleotest Bio Ltd., Budapest, Hungary) was added to the gel and 1×TBE buffer was used for the gel electrophoresis. A 100 bp ladder (GeneRuler™ 100 bp Plus DNA Ladder, Biocenter Ltd., Szeged, Hungary) was used as molecular weight marker. The bands were visualised with FluorChem M system (Bio-Science Ltd., Budapest, Hungary).

### 2.8. Statistical Analysis

The averages and standard deviations (SD) were calculated with SPSS v.22.0 [40] software which was also used for the logarithmic transformation (log_10_) of the amounts and variance analysis.

## 3. Results

### 3.1. Enumeration and Isolation of Staphylococcus aureus

**Figure 1:**
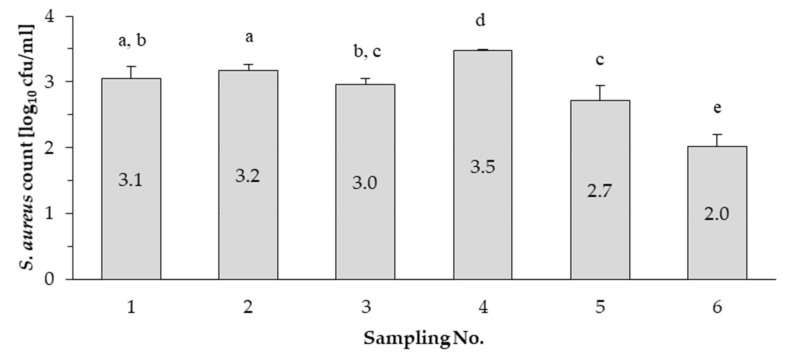
The mean *S. aureus* count in bulk milk during the control program. The means marked with the same letters (a, b, c, d or e) do not differ significantly from each other (*p* > 0.05).

Based on the results, *S. aureus* occurred in all bulk tank milk samples collected during this study. The mean values ranged from 2.0 to 3.5 log_10_ cfu/mL (Figure 1). In the study of Peles et al. [41] the mean *S. aureus* count was <2.7 log_10_ cfu/mL in the same (LF5) farm. In this study the *S. aureus* count in the samples collected during the sixth sampling (in December; 2.0 log_10_ cfu/mL) was lower (*p* < 0.05) than in the samples collected during all the other sampling occasions, including the first sampling (3.1 log_10_ cfu/mL), which was also performed in winter (February).

On the other hand, the results also show that the *S. aureus* count decreased by the end of the year, the amounts did not exceed the refusal limit (M = 2.7 log_10_ cfu/mL) set in the regulation of the Hungarian Ministry of Health 4/1998 (XI.11) in the case of the last two samplings. 

Seventeen *S. aureus* strains were chosen (two to four strains from each sampling occasion) from bulk tank milk samples for further investigations. Fifteen strains (88%) were dark grey, and two strains (12%) were black on BPA and there were clear zones around the colonies. These results are consistent with the results of Peles et al. [41], because the six *S. aureus* isolates collected from the same farm (LF5) by the authors had similar properties.

In the case of all the 17 *S. aureus* strains isolated from bulk milk, the latex agglutination test was positive and the presence of *nuc* gene was revealed.

### 3.2. Identification of Staphylococcus aureus with MALDI-TOF MS

Table 2 shows the results of the identification obtained with the MALDI-TOF MS and the results of the *spa* typing. Based on the results (best score and best-matching isolate) of the tests with the MALDI-TOF MS device, all strains were confirmed as *S. aureus* and the isolates, except for the SA57A, SA57B and SA57C, matched the spectra of three *S. aureus* reference strains (1: *S. aureus* subsp. *aureus* DSM 20231T; 2: *S. aureus* subsp. *aureus* DSM 799; 3: *S. aureus* ATCC 33862 THL) in the Bruker MALDI Biotyper database. All isolates (except for SA57B) gave MS best scores ≥ 2.300, so the species were reliably identified. In the case of SA57B, the MS best score was 2.270, so the genus identification was secure, but the species identification was only probable.

### 3.3. Spa Typing

Two *spa* types (t164 and t1987) were identified among the 17 isolates originated from the bulk milk samples (Table 2). The t164 *spa* type had eight repeats, and the t1987 *spa* type had five repeats. The *spa* types detected in this study were singletons according to the BURP algorithm of the Ridom Staphtype software.

**Table 2:** Identification of isolates by MALDI-TOF MS and the results of the *spa* typing.

Strain ID	Sampling No.	Best Score	Organism (Best Match) *	*spa* Type	*spa* Repeats
SA33	1.	2.361	1	t164	r07r06r17r21r34r34r22r34
SA34	1.	2.374	1	t164	r07r06r17r21r34r34r22r34
SA35A	1.	2.369	1	t164	r07r06r17r21r34r34r22r34
SA35B	1.	2.374	2	t164	r07r06r17r21r34r34r22r34
SA39A	2.	2.342	1	t164	r07r06r17r21r34r34r22r34
SA39B	2.	2.312	3	t164	r07r06r17r21r34r34r22r34
SA44	3.	2.419	2	t164	r07r06r17r21r34r34r22r34
SA45	3.	2.359	1	t164	r07r06r17r21r34r34r22r34
SA53A	4.	2.318	1	t164	r07r06r17r21r34r34r22r34
SA53B	4.	2.410	1	t164	r07r06r17r21r34r34r22r34
SA53D	4.	2.305	2	t164	r07r06r17r21r34r34r22r34
SA54A	5.	2.302	2	t164	r07r06r17r21r34r34r22r34
SA54B	5.	2.371	2	t164	r07r06r17r21r34r34r22r34
SA54C	5.	2.500	1	t1987	r07r06r17r21r34
SA57A	6.	2.333	-	t164	r07r06r17r21r34r34r22r34
SA57B	6.	2.270	-	t164	r07r06r17r21r34r34r22r34
SA57C	6.	2.417	-	t164	r07r06r17r21r34r34r22r34

* 1: *S. aureus* subsp. *aureus* DSM 20231T; 2: *S. aureus* subsp. *aureus* DSM 799; 3: *S. aureus* ATCC 33862 THL.

### 3.4. Haemolysis Testing

A total of 14 of the 17 studied strains (82%) showed β-haemolysis on blood agar, 2 of the 17 strains (12%) had incomplete haemolytic phenotype (expressed double zone) and 1 strain (6%) showed weak β-haemolysis. Table 3 shows the characteristics (tellurite reduction, lecithinase activity, haemolysis type, antibiotic susceptibility, enterotoxin gene harbouring ability) of *S. aureus* strains isolated from bulk milk.

### 3.5. Antibiotic Susceptibility Testing

Based on the results, all strains were susceptible to the antibiotic agents tested, except for the penicillin G (Table 3). 

### 3.6. Staphylococcal Enterotoxin-Encoding Genes

All 17 strains were positive for one or more enterotoxin-encoding genes. Among the new types of SEs and SEls, five different enterotoxin-encoding genes were identified. A total of 11 out of 17 isolates (65%) were found to harbour *seg*, *sei*, *selm*, *seln*, *selo* genes; 4 of the 17 strains (24%) harboured *sei*, *selm*, *seln*, *selo* genes and 2 strains (11%) harboured the *sei* gene alone (Table 3). During the first and the last two samplings, it was found that different enterotoxin-producing strains could be isolated from the collected milk samples originated from the same milk tank in each sampling occasions.

**Table 3:** Characteristics of *S. aureus* strains isolated from bulk milk.

Strain ID	Sampling No.	Characteristics				
		Tellurite Reduction	Lecithinase Activity	Hemolysis	Antibiotic Resistance *	Detected Enterotoxin Genes
SA33	1.	dark grey	+	β	R (P)	*sei*
SA34	1.	dark grey	+	β	R (P)	*seg, sei, selm, seln, selo*
SA35A	1.	dark grey	+	β	R (P)	*seg, sei, selm, seln, selo*
SA35B	1.	dark grey	+	β	R (P)	*seg, sei, selm, seln, selo*
SA39A	2.	dark grey	+	β	R (P)	*sei, selm, seln, selo*
SA39B	2.	dark grey	+	β	R (P)	*sei, selm, seln, selo*
SA44	3.	dark grey	+	β	R (P)	*seg, sei, selm, seln, selo*
SA45	3.	dark grey	+	β	R (P)	*seg, sei, selm, seln, selo*
SA53A	4.	dark grey	+	β	R (P)	*seg, sei, selm, seln, selo*
SA53B	4.	dark grey	+	β	R (P)	*seg, sei, selm, seln, selo*
SA53D	4.	dark grey	+	β	R (P)	*seg, sei, selm, seln, selo*
SA54A	5.	dark grey	+	α + β	R (P)	*seg, sei, selm, seln, selo*
SA54B	5.	dark grey	+	α + β	R (P)	*sei, selm, seln, selo*
SA54C	5.	dark grey	+	weak β	R (P)	*sei*
SA57A	6.	dark grey	+	β	R (P)	*seg, sei, selm, seln, selo*
SA57B	6.	black	+	β	R (P)	*sei, selm, seln, selo*
SA57C	6.	black	+	β	R (P)	*seg, sei, selm, seln, selo*

* R: resistant; P: penicillin G.

## 4. Discussion

According to Zeinhom et al. [42] the somatic cell count and the amount of pathogen microorganisms may increase in summer milk samples and this can be related to the heat stress of cows. Due to heat stress caused by unfavourable weather conditions the incidence of udder infections, such as *S. aureus* infections can be increasing. In our research, the fourth sampling occasion was in summer, and this may explain the increased *S. aureus* count (3.5 log_10_ cfu/mL) in bulk milk. It can also be stated that the *S. aureus* count decreased by the end of our research, and this is a possible outcome of the *S. aureus* control program.

The most common *spa* type was t164; 16/17 strains (94%) belonged to this type. The *spa* type of the SA54C strain was t1987, which differed from the t164 only in three *spa* repeats (r34r22r34). Therefore,, the t164 *spa* type occurred most frequently, the strain with t1987 *spa* type only occurred while testing the raw milk samples collected during the fifth sampling occasion. In their research, Hwang et al. [43] assorted the similar *spa* types (t164 and t1987) into one cluster and stated that evolutionary relationship can be displayed in each cluster between the isolates. However, unlike their study, we found that more than one *spa* type (t164 and t1987) was detected in a single dairy farm.

In the study of Peles et al. [41] three of the six strains (50%) isolated from bulk tank milk from LF5 farm showed weak haemolysis, two of the six strains (33%) isolated from udder quarter milk showed weak haemolysis and one strain (17%) originated from the same origin showed α-haemolysis on blood agar. These results are different from the results of our study. In the study of Morandi et al. [44], 50 of the 81 *S. aureus* strains (62%) isolated from dairy products made from cow milk showed β-haemolysis, while 29 strains (36%) showed double (α + β) haemolysis and α-haemolysis was occurred in the case of only two (2%) cow isolates. From the 148 *S. aureus* strains isolated from various food products in the study of Pereira et al. [33] 81% showed β-haemolysis, 11% showed γ-haemolysis and eight percent showed α-haemolysis on blood agar.

Peles et al. [41] also experienced in their study, that all *S. aureus* isolates collected from the LF5 dairy farm were resistant to penicillin G but were susceptible to the other antibiotics tested (methicillin; cefoxitin; lincomycin; tetracycline; erythromycin; trimethoprim/sulfamethoxazole). Similar results were published previously by other authors: isolates investigated by Abo-Shama [45] were susceptible to cefoxitin, Visciano et al. [46] published strains which were susceptible to gentamicin. In the study of Morandi et al. [44] none of the tested 81 strains isolated from cow dairy products showed resistance to methicillin. Pereira et al. [33] obtained that the isolates collected from bovine mastitis and raw cow’s milk were demonstrated to be the most susceptible to the tested antibiotics (erythromycin, gentamicin, tetracycline, chloramphenicol, ciprofloxacin, rifampicin, ampicillin, penicillin, oxacillin, vancomycin, nitrofurantoin). Among the *S. aureus* isolated from raw milk by André et al. [47] 71% were resistant to penicillin and 33% were resistant to tetracycline.

Food poisoning is most commonly caused by the classical enterotoxins SEA and SEB, and by the newly described enterotoxin SEH [14,15]. None of the strains examined in this study harboured enterotoxin genes encoding these enterotoxins. According to Umeda et al. [16] new SE/SEls (e.g., *seg*, *sei*, *selm*, *seln*, *selo* and *selu*) can also be potential causes of foodborne outbreaks. In this study, all the strains were found to harbour at least one of these genes; therefore, it is essential in the dairy farm to decrease the occurrence and keep the prevalence of *S. aureus* low, and the implementation of the *S. aureus* control program is highly justified, as well. However, it is worth noting that the *S. aureus* concentration of at least 10^5^ cfu/mL in milk could produce enough enterotoxins to cause food poisoning [48] but this number of bacteria was not present in our samples. In the study of Peles et al. [41] two of the three *S. aureus* strains isolated from bulk tank milk harboured *seb* gene. One of the three strains isolated from udder quarter milk harboured *seg* and *sei* genes. The third strain isolated from bulk milk and the two other strains isolated from udder quarter milk did not harbour any of the tested enterotoxin genes (*sea*, *seb*, *sec*, *sed*, *see*, *seg*, *seh*, *sei*, *sej*, *tsst*). Morandi et al. [44] stated that 58 of the 81 *S. aureus* strains (72%) from dairy products made from cow milk were positive for *se*. *Sea*, *sed*, and *sej* were found more densely. Only two strains (2%) (isolated from milk and from cheese) were found to have the *sec* gene and two strains (2%) harboured *seg* and *sei* genes. In the study of Pereira et al. [33] seven of the 20 *S. aureus* strains (35%) isolated from raw cow’s milk harboured *se*. Three strains (43%) harboured *seg* and *sei*, two strains (29%) harboured *sec* (bovine), and two strains (28%) harboured *sec* (bovine) and *seg* genes. Karahan et al. [49] investigated SE genes (classical *se*-s and *seg*, *seh*, *sej*, *sei*) by multiplex PCR method in *S. aureus* strains isolated from bovine mastitis and found that 27 of the strains (29%) harboured one or more *se* genes, and also *sei* was the most common. In the study of Korpysa-Dzirba and Osek [50] five out of 66 isolates (7.6%) of *S. aureus* isolated from raw cow’s milk carried genes encoding classical SE-s. Of the 20 virulence genes investigated by Dai et al. [51] in the case of *S. aureus* strains isolated from pasteurised milk, *seg*, *sei*, and *sem* were detected the most frequently showing 41.7% of prevalence. *sea*, *seb*, *sed*, *see*, *seu*, *seq*, *sej*, *ser*, *sek*, and *pvl* were not detected, but *sen*, *sec*, *sel*, *seo*, *sep*, *seh*, and *tsst* were detected in the isolates.

Hwang et al. [43] found in their study that most of the isolates (seven of eight) with t164 *spa* type and three of the seven isolates with t1987 *spa* type harboured *seg*, *sei*, *selm*, *seln* and *selo* genes. Similarly, in our study, 11 of the 17 isolates with t164 *spa* type harboured these genes without harbouring classical enterotoxin genes. The isolate with t1987 *spa* type harboured only *sei* gene.

## 5. Conclusions

The results show that the mean *S. aureus* count decreased by the end of the year, and the amounts did not exceed the refusal limit in the case of the last two samplings. However, in winter lower amounts of pathogenic bacteria (e.g., coliform bacteria) can be detected in milk [52,53], the first sampling occasion was in winter similarly to the last sampling occasion and yet a significantly higher mean *S. aureus* count could be detected in milk than that obtained during the test of the samples collected in the last sampling occasion. Thus, the decreasing *S. aureus* count can be attributed to the effectiveness of the control program. Furthermore, based on our results, *S. aureus* strains with different characteristics could be detected from raw milk of a single dairy farm. Two different *spa* types (t164 and t1987) were identified among the 17 strains isolated in the dairy farm. The t1987 *spa* type isolate was detected during the tests of the milk samples collected during the fifth sampling occasion, but it could not be detected in the sixth sampling occasion. Differences between strains were also reflected in the diversity of haemolysis types, although there was no difference between strains in terms of antibiotic susceptibility. All strains were susceptible to most of the antibiotics tested but were resistant to penicillin G. None of the isolates harboured the classical enterotoxin genes, but all the strains were found to harbour at least one of the *seg*, *sei*, *selm*, *seln* and *selo* genes. Since the new SEs/SEls can also be potential cause of foodborne outbreaks, it is essential in the dairy farm to decrease the occurrence and keep the prevalence of *S. aureus* low. 

## Figures and Tables

**Table 1:** Primers for amplification of genes encoding staphylococcal enterotoxins and thermonuclease.

Enterotoxin genes	Primers	Nucleotide Sequences (5′-3′)	Amplification Size (bp)	Reference
*sea*	GSEAR-1	GGTTATCAATGTGCGGGTGG	102	[36]
	GSEAR-2	CGGCACTTTTTTCTCTTCGG		
*seb*	GSEBR-1	GTATGGTGGTGTAACTGAGC	164	[36]
	GSEBR-2	CCAAATAGTGACGAGTTAGG		
*sec*	GSECR-1	AGATGAAGTAGTTGATGTGTATGG	451	[36]
	GSECR-2	CACACTTTTAGAATCAACCG		
*sed*	GSEDR-1	CCAATAATAGGAGAAAATAAAAG	278	[36]
	GSEDR-2	ATTGGTATTTTTTTTCGTTC		
*see*	SA-U	TGTATGTATGGAGGTGTAAC	213	[37]
	SA-E rev	GCCAAAGCTGTCTGAG		
*seg*	SEG-F	GTTAGAGGAGGTTTTATG	198	[38]
	SEG-R	TTCCTTCAACAGGTGGAGA		
*seh*	SEH-F	CAACTGCTGATTTAGCTCAG	173	[38]
	SEH-R	CCCAAACATTAGCACCA		
*sei*	SEI-F	GGCCACTTTATCAGGACA	328	[38]
	SEI-R	AACTTACAGGCAGTCCA		
*sej*	SEJ-F	GTTCTGGTGGTAAACCA	131	[38]
	SEJ-R	GCGGAACAACAGTTCTGA		
*selm*	SEM-F	CATATCGCAACCGCTGA	148	[38]
	SEM-R	TCAGCTGTTACTGTCGA		
*seln*	SEN-F	GGCAATTAGACGAGTCA	237	[38]
	SEN-R	ATCGTAACTCCTCCGTA		
*selo*	SEO-F	GTCAAGTGTAGACCCTA	288	[38]
	SEO-R	TGTACAGGCAGTATCCA		
*ser*	SER1-F	AGATGTGTTTGGAATACCCTAT	123	[39]
	SER2-R	CTATCAGCTGTGGAGTGCAT		
*nuc*	NUC-F	GCGATTGATGGTGATACGGTT	270	[31]
	NUC-R	AGCCAAGCCTTGACGAACTAAAGC		

## Data Availability

The data presented in this study are available within the article.
